# Cryptochrome-mediated blue-light signal contributes to carotenoids biosynthesis in microalgae

**DOI:** 10.3389/fmicb.2022.1083387

**Published:** 2022-12-22

**Authors:** Zhongyi Zhang, Tianli Han, Jikang Sui, Hui Wang

**Affiliations:** ^1^Solar Energy Laboratory, Qingdao Institute of Bioenergy and Bioprocess Technology, Chinese Academy of Sciences (CAS), Qingdao, China; ^2^Shandong Energy Research Institute, Qingdao, China

**Keywords:** cryptochrome, blue light, carotenoid biosynthesis, *Phaeodactylum tricornutum*, *Haematococcus pluvialis*

## Abstract

Microalgae are considered as ideal cell factories for producing natural carotenoids which display favorable biological activities. As the most important abiotic factor, light not only provides energy for photosynthetic metabolism, but also regulates numerous biological processes. Blue light is the main wavelength of light that can travel through water. Previous studies have shown that blue light triggered carotenoid accumulation in several microalgae species, but the molecular mechanism remains unclear. Cryptochromes were blue-light-absorbing photoreceptors that have been found in all studied algal genomes. In this study, several different types of cryptochrome genes were cloned from *Haematococcus pluvialis* and *Phaeodactylum tricornutum*. Among them, cryptochrome genes *HpCRY4* from *H*. *pluvialis* and *PtCPF1* from *P*. *tricornutum* were upregulated under blue light treatment, in correlation with the increase of astaxanthin and fucoxanthin contents. Besides, heterologous expression and gene knockout was performed to verify the function of *HpCRY4* and *PtCPF1* in regulating carotenoid biosynthesis in microalgae. These results indicate that carotenoid biosynthesis in microalgae promoted by blue light was mediated by cryptochromes as photoreceptors.

## Introduction

Microalgae have drawn great attention for their ability to produce a wide range of high-value-added compounds, such as proteins, carbohydrates, carotenoids and lipids, for healthy, feed additives, fuel production, and drug manufacturing (Pulz and Gross, 2004; Chacón-Lee and González-Mariño, 2010; Borowitzka, 2013). Among them, carotenoids are widely present in photoautotrophic organisms, functioning in photosynthetic light harvesting, and protecting photosynthetic apparatus against reactive oxygen species (ROS) generation (Frank and Cogdell, 1996; Hashimoto et al., 2016). Recently, carotenoids such as fucoxanthin and astaxanthin have broad commercial prospects in the fields of nutraceutical and pharmaceutical manufacturing (Christaki et al., 2013; Galasso et al., 2017; Ávila-Román et al., 2021), making microalgae the main source for commercial carotenoids production (Dufossé et al., 2005).

Light is not only an essential source of energy, but also an important environmental signal to regulate biochemical, physiological, and behavioral processes (Spetea et al., 2014). Light quality can trigger the behavior and developmental responses of photosynthetic organisms (Mittag and Wilhelm, 2017). Blue light is the main wavelength of light that can travel through seawater and plays an important role in adapting algae to changing environmental conditions (Depauw et al., 2012). Previous studies have shown that blue light helps to increase the accumulation of carotenoids in microalgae (Katsuda et al., 2004; Lababpour et al., 2004; Suyono et al., 2015; Wang et al., 2018). Meanwhile, the expression levels of key genes involving carotenoid biosynthesis (*ZEPs*, *PSY*, *et al*) were proved to be significantly regulated by blue light strengthening (Valle et al., 2014; Yang and Wei, 2020). Although many studies demonstrated a correlation between blue light and carotenoid biosynthesis, our knowledge about the exact mechanisms is still limited.

Cryptochromes (CRYs), as blue-light-absorbing photoreceptors in plants and animals, play a critical regulatory role in plant development and the entrainment of circadian rhythms (Hoang et al., 2008; Chaves et al., 2011; Rosensweig et al., 2018). Based on evolutionary analysis, cryptochromes originated from the ancient blue-light-dependent DNA repair enzymes called photolyases, but have lost DNA repair activity and acquired a novel role in signaling (Lin, 2002; Sancar, 2003; Chaves et al., 2011). Cryptochromes were first found in *Arabidopsis thaliana* and subsequently identified ubiquitously in animals, prokaryotes, and eukaryotes. Especially, cryptochromes have been found in all studied algal genomes available so far. Algae mainly live in aquatic environments where the blue wavelengths are the dominant light components. The cryptochromes may play an important role in light signal transduction in algae. Therefore, it is reasonable to speculate that cryptochromes mediate the regulation of carotenoid biosynthesis under blue light treatment.

In this study, *Phaeodactylum tricornutum* and *Haematococcus pluvialis* cells were cultured under white and blue light, respectively. And the full-length cDNA of cryptochrome genes were cloned from *P*. *tricornutum* and *H*. *pluvialis*. The relationship between the transcription level of cryptochrome genes and important carotenoid contents in *P*. *tricornutum* and *H*. *pluvialis* was analyzed to identify the target cryptochrome genes responding to blue light. In addition, heterologous expression and gene knockout of the target cryptochrome genes were performed in *P*. *tricornutum* to further verify the function of regulating carotenoid accumulation. This study provides a new perspective to explain how blue light promotes carotenoid biosynthesis in microalgae.

## Materials and methods

### Strains and culture conditions

The *P*. *tricornutum* strain used in this study, UTEX 646, was obtained from the Culture Collection of Algae at the University of Texas at Austin (UTEX). The diatoms were maintained in 50 ml Erlenmeyer flasks containing 20 ml 2f liquid medium. The 2f medium contained (per liter): 300 mg NaNO_3_, 80 mg Na_2_SiO_3_·9H_2_O, 20 mg NaH_2_PO_4_·H_2_O, 4.36 mg Na_2_EDTA·2H_2_O, 3.15 mg FeCl_3_·6H_2_O, 9.8 μg CuSO_4_·5H_2_O, 22 μg ZnSO_4_·7H_2_O, 189 μg MnCl_2_·4H_2_O, 7 μg Na_2_MoO_4_·2H_2_O, 12 μg CoCl_2_·6H_2_O. The medium was prepared with filtrated seawater and autoclaved at 121°C for 20 min. Cultures were kept at 23 ± 1°C, with a continuous irradiance of 30 μmol m^−2^ s^−1^. The solid 2f medium for transformants selection was prepared by supplementing 1% (w/v) agar (Solarbio, China). After autoclaving, the medium was cooled to 60°C and mixed with 100 μg mL^−1^ zeocin before distribution into Petri dishes.

The *H*. *pluvialis* strain, SCCA-PK0084, was obtained from the Scandinavian Culture Center for Algae and Protozoa (SCCAP) at the University of Copenhagen, Denmark. The *H*. *pluvialis* culture was maintained in 50 ml flasks containing 20 ml sterilized BG11 medium at 23 ± 1°C and continuously illuminated with 30 μmol m^−2^ s^−1^ of light.

To evaluate the light-induced effects on the biosynthesis of carotenoids, the algal cells were grown to the mid-logarithm growth phase in 100 ml bubble columns (30 cm in height, 4 cm in diameter, 100 ml medium) with CO_2_-enriched air (1.5%, v/v) flowing at a rate of 30 l h^−1^. After that, the cells were placed without light for 48 h for dark treatment, and then they were treated with different lights (white and blue) of the same light intensity (5 ~ 6 μmol m^−2^ s^−1^), respectively (Supplementary Figure S1). The microalgal cells at different time points were collected by centrifugation at 10,000 rpm for 5 min, and the pellets were stored at −80°C for subsequent pigments and transcription analysis.

### Pigments extraction and analysis

Total pigments of *P*. *tricornutum* cells were extracted from dried samples with organic solvents and analyzed by the high-performance liquid chromatography (HPLC) method (Mulders et al., 2015; Chen et al., 2017; Yang and Wei, 2020). Briefly, 30 mg of freeze-dried *P*. *tricornutum* cells were ground into powder and completely mixed with 2 ml pre-cooled methanol/acetone (1:1, v/v). The supernatant was collected by centrifugation at 12,000 rpm for 15 min. Repeat addition of the organic solvents and centrifugation until the pellet was colorless. All supernatants were collected and dried after filtering through a 0.22 μm nylon membrane for further pigment analysis.

The pigments were analyzed through an Agilent 1,200 HPLC system (Waters, United States) equipped with an Agilent ZORBAX Eclipse XDB-C18 chromatographic column (5 μm particle size, 250 × 4.6 mm; Agilent, United States). The mobile phase was 85% methanol and 100% ethyl acetate with a flow rate of 0.8 ml min^−1^. The ratio increased from 100:0 to 30:70 over 16 min, maintained at 30:70 for 9 min, and then decreased back to100:0 over 10 min. The chromatogram was recorded at 450 nm.

Total pigments of *H*. *pluvialis* were extracted and analyzed based on the above approach with a few adjustments. Cold methanol/chloroform (2:1, v/v) was used as the organic solvent. Chloroform and double distilled water (DDW) were added into the collected supernatants until the volume ratio of chloroform: methanol: water was 10: 10: 9. The lower fluid containing pigment and chloroform was collected by centrifugation at 8,000 rpm for 5 min and filtered through a 0.22 μm membrane. The mobile phase of HPLC was 100% methanol and 100% acetonitrile, and their ratio was 75:25. The chromatogram was recorded at 480 nm.

### Cloning of cryptochrome genes from microalgae

Genome-wide analysis was performed to search the cryptochrome genes in *P*. *tricornutum* and *H*. *pluvialis* genomes based on the conserved domain of cryptochrome. And then, specific primers were designed to amplify the full-length cDNA of cryptochrome genes in *P*. *tricornutum* and *H*. *pluvialis*. All primers were listed in Supplementary Table S1.

Total RNA was extracted from algal cells using SparkZol Reagent AC0101 (Shandong Sparkjade Biotechnology Co., Ltd., China) according to the protocol. One μg of total RNA each sample was reversely transcribed into cDNA using Evo M-MLV II (Accurate Biotechnology, China) according to the manufacturer’s instructions. The cDNA was diluted to 10 ng μL^−1^ in sterile distilled water (SDW) for PCR template or mRNA abundance quantification. The full-length cDNAs of cryptochrome genes were amplified using PrimeSTAR HS DNA polymerase (Takara, Japan) with specific primers. The PCR products were analyzed by 1% agarose gel electrophoresis, and the clear and bright bands with the correct length were recycled and ligated with pCE2 vector. The successful ligated product was introduced into *E*. *coli* for sequencing.

The microalgal cryptochrome protein sequences were translated according to the cDNA sequences by ORF finder and aligned with some typical cryptochromes using ClustalW 1.83 (Chenna et al., 2003). The typical cryptochrome sequences were available in GenBank^1^ and their IDs were listed in Supplementary Table S2. The phylogenic tree was inferred from the protein alignments using MEGAX 10.1.8 (Tamura et al., 2007) with the neighbor-joining method (Poisson model; 1,000 bootstrap replicates).

### Quantitative real time PCR

Quantitative real-time PCR (qRT-PCR) was performed on the Roche Light Cycler 480 (Roche, Switzerland) with PowerUp SYBR Green Master Mix (Thermo Fisher Scientific, United States) according to the manual, and the data were collected and analyzed using 2^−^^∆∆Ct^ method (Livak and Schmittgen, 2001) by the Excel software. Housekeeping genes *β-actin* and *α-tubulin* were used as the internal control in *P*. *tricornutum* and *H*. *pluvialis*, respectively. All primers used for qRT-PCR were listed in Supplementary Table S3. All assays were performed three times, and a reaction without reverse transcriptase was used as a negative control.

### Vector construction

In-Fusion cloning technology was used to construct the vectors for gene heterologous expression. Firstly, the pPha-T1 (Zaslavskaia et al., 2001) plasmid was digested with *Eco*R I and *Xba* I restriction enzymes at 37°C for 30 min. The long fragments containing plasmid backbone were recycled through electrophoresis. The full-length cDNA of cryptochrome genes were amplified by PCR with specific primers (Supplementary Table S4) and the 5′ and 3′ ends of the cDNA were made to have the same sequence as the ends of the linearized pPha-T1 vector. After that, the full-length cDNA of cryptochrome genes and the linearized pPha-T1 vector were ligated using Vazyme ClonExpress Ultra One Step Cloning Kit (Vazyma, China) according to the manual.

CRISPR-Cas9 was used to knock out the cryptochrome gene. Guide RNAs (gRNAs) were designed by the online design tool crispor.tefor.net (Haeussler et al., 2016) based on the PAM sequence (5′-NGG-3′) in the exon region of the target gene. Then the target oligonucleotides were annealed at 95°C for 5 min, and slowly cooled down at room temperature to form the double-stranded inserts which were ligated into the *Bsa*I digested vector PtPuc3_diaCas9_sgRNA.^2^ The PtPuc3_diaCas9_sgRNA vector contains a diatom-codon optimized Cas9 expression cassette fused with the *P*. *tricornutum* LHCF2 promoter and LHCF1 terminator, and a single-guide RNA (sgRNA) cassette driven by the *P*. *tricornutum* U6 promoter (Nymark et al., 2016). A *Ble* gene (Apt et al., 1996) controlled by the LHCF11 promoter and LHCF1 terminator was in the backbone of vector PtPuc3_diaCas9_sgRNA which confers resistance to the antibiotic zeocin.

### Genetic transformation of *P. tricornutum*

The biolistic transformation was used to deliver DNA into *P*. *tricornutum* cells. The microparticle bombardment method was performed according to published procedures with minor modifications (Apt et al., 1996). Tungsten particles (0.7 μm diameter, BioRad) were coated with DNA in the presence of 2.5 M CaCl_2_ and 0.1 M spermidine. *P*. *tricornutum* cells in the exponential growth phase were collected and spread on the 2f agar plates. The bombardment was performed by a PDS-1000/He Biolistic Particle Delivery system (Bio-Rad, United States) fitted with 1,350 psi rupture discs. Bombarded cells were collected and suspended in liquid 2f medium, and then placed without light 24 h for recovery; the suspension was plated onto solid 2f medium containing 100 μg mL^−1^ zeocin. The plates were placed at 23 ± 1°C under a continuous irradiance of 30 μmol m^−2^ s^−1^ light intensity for 2–3 weeks until the resistant colonies were visible and transferable.

### Statistical analysis

All data were performed as the average of biological triplicate and shown as mean ± standard deviation (SD). GraphPad_Prism V8.4.0 software was used to plot histograms and line charts. The statistical analysis was performed by Student’s t-test with SPSS V24.0 statistical software (IBM, United States), and significant differences are marked with lowercase letters (*p* < 0.05).

## Results and discussion

### Blue light promotes carotenoid biosynthesis in algae

Excess light can lead to photo-inhibition and reduce cell growth (Difusa et al., 2015). To avoid such a scenario, a low light of 5 ~ 6 μmol m^−2^ s^−1^ was used as a signal to induce carotenoid biosynthesis. The carotenoid contents of *P*. *tricornutum* and *H*. *pluvialis* were determined by HPLC after 300 min under different light treatments, including dark, white and blue.

**Figure 1:**
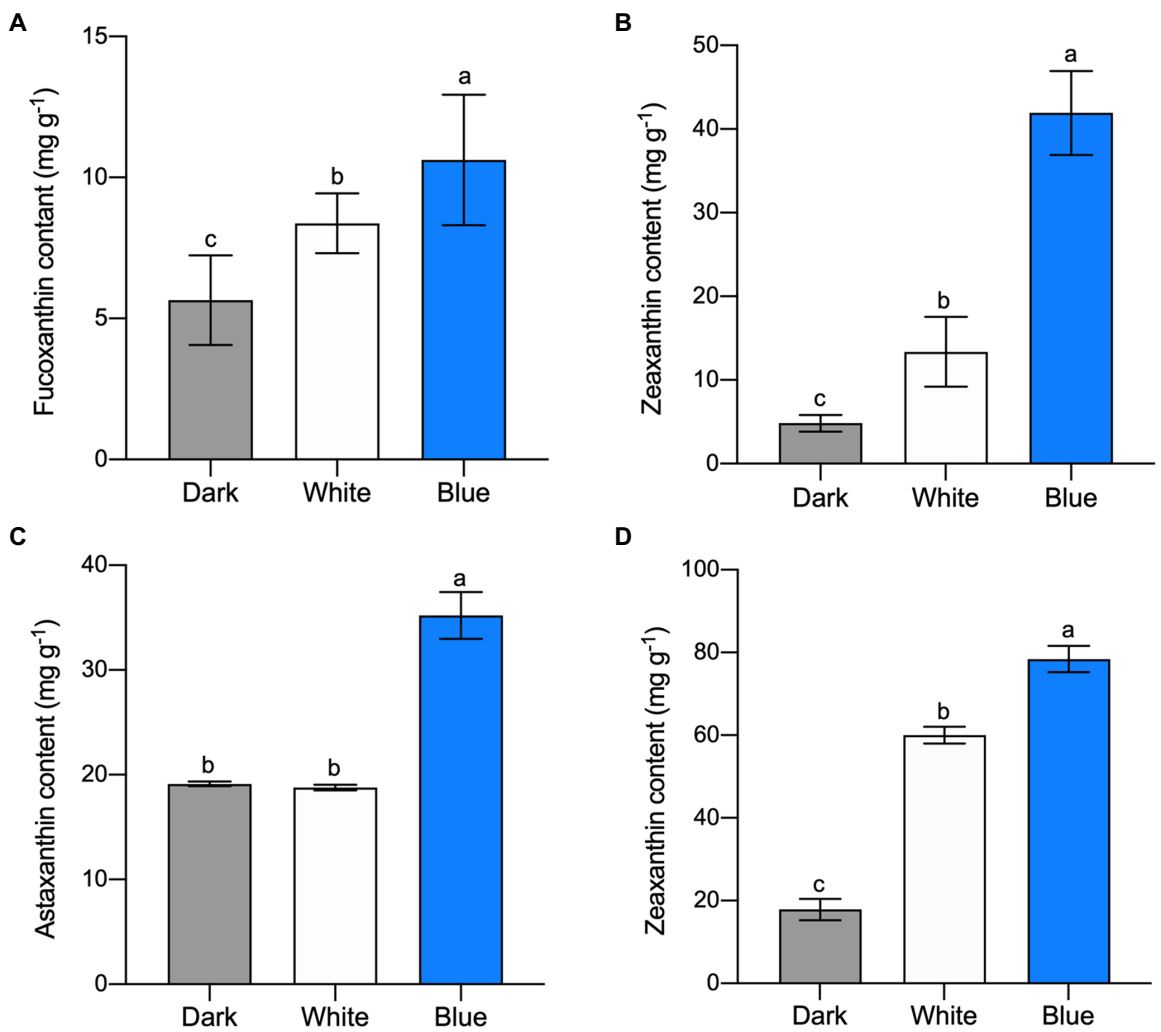
Carotenoid content in microalgae under different light irradiations. Fucoxanthin **(A)** and zeaxanthin **(B)** content in *P*. *tricornutum* under different light after 300 min, respectively. Astaxanthin **(C)** and zeaxanthin **(D)** content in *H*. *pluvialis*, respectively. The vertical bars are the means ± SD of three biological replicates and lowercase letters indicate significant differences by Student’s *t*-test (*p* < 0.05).

Fucoxanthin and zeaxanthin content of *P*. *tricornutum* cells were significantly increased after white and blue light irradiation. Specifically, fucoxanthin content reached 8.38 and 10.65 mg g^−1^ under white and blue light, respectively (Figure 1A). It was 26.73 and 88% higher than that treated with dark (5.65 mg g^−1^). Zeaxanthin content was 24.26 and 41.91 mg g^−1^ under white and blue light which was much higher than that under dark (4.83 mg g^−1^; Figure 1B). Although these two carotenoids’ contents were higher under both light irradiations, we can see that blue light was much more conducive to carotenoid accumulation in *P*. *tricornutum*. The same carotenoid content promotion occurred in *H*. *pluvialis*. Astaxanthin content under blue light reached 35.21 mg g^−1^ which was 84.06% higher than that under dark (Figure 1C). Meanwhile, zeaxanthin, as a former of astaxanthin, reached 60.01 and 78.42 mg g^−1^ under white and blue light respectively, much higher than that under dark (Figure 1D). The above results indicate that blue light irradiation promotes the accumulation of carotenoids, such as fucoxanthin, zeaxanthin and astaxanthin, in algae.

In consist with our study, blue light promoted astaxanthin accumulation in *H*. *pluvialis* had been reported in previous studies (Katsuda et al., 2004; Lababpour et al., 2004; Suyono et al., 2015). Besides the above carotenoids we analyzed and discussed in the present study, many other carotenoid accumulations are also related to blue light. For example, blue light elevated the beta-carotene biosynthesis in *Dunaliella salina* (Fu et al., 2013; Han et al., 2019); higher blue light intensities help to achieve the carotenoid and xanthophyll pigments enrichment in mustard, beet and parsley (Samuolienė et al., 2017). It seems that the accumulation of carotenoids induced by blue light irradiation is ubiquitous in higher plants and algae.

### Identification of cryptochromes from *P. tricornutum and H. pluvialis*

**Figure 2:**
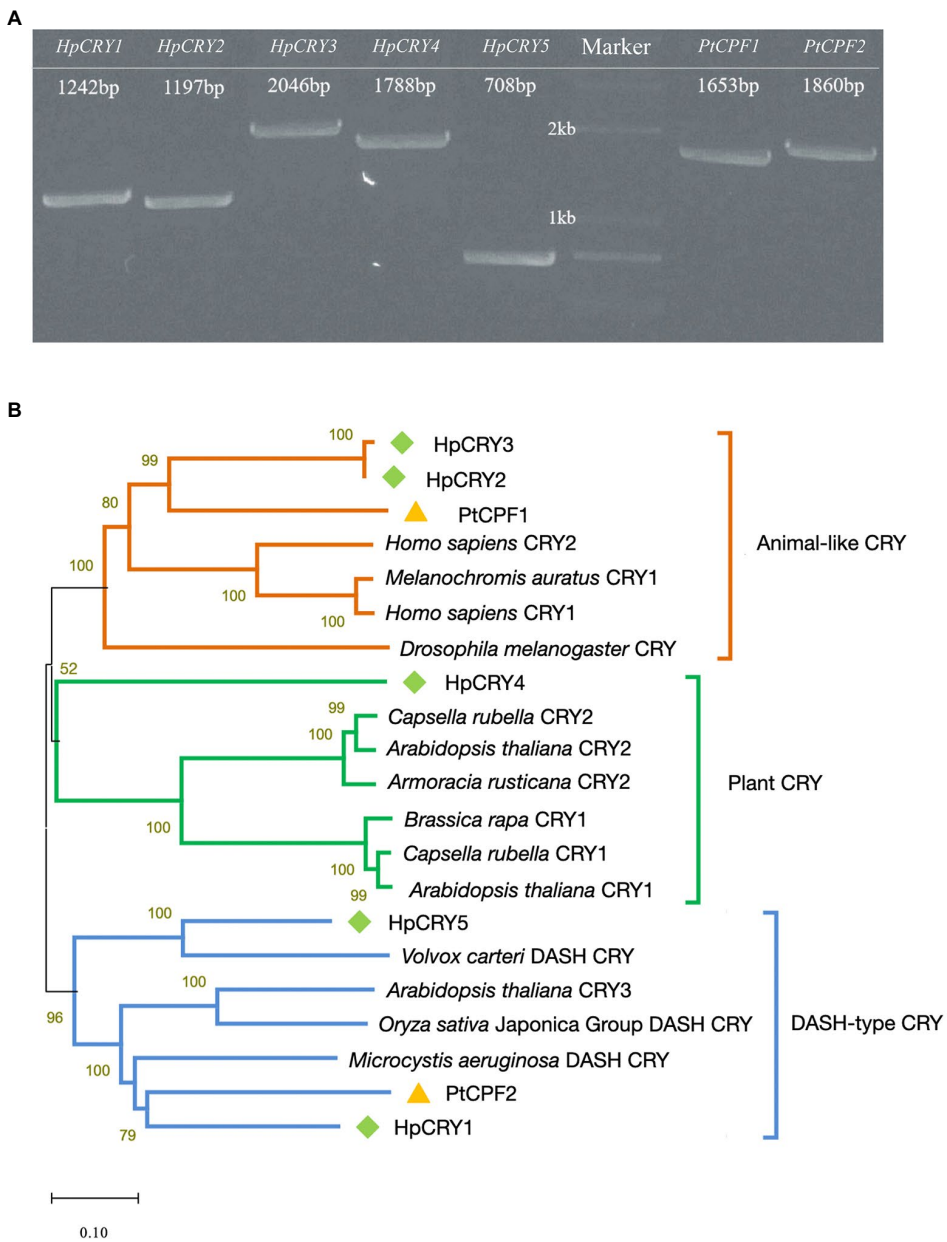
Identification of cryptochromes in *P*. *tricornutum* and *H*. *pluvialis*. **(A)** Full-length cDNA cloning of the cryptochrome genes from *P*. *tricornutum* and *H*. *pluvialis*. The numbers in panel **A** indicate the length of the sequences. **(B)** Phylogenic tree based on amino acid sequences of cryptochromes.

As important photoreceptors for positive responses to blue light and near-ultraviolet light treatment, cryptochromes might play a role in carotenoid accumulation regulation. Therefore, genome-wide analysis was performed to search the cryptochrome genes in *P*. *tricornutum* and *H*. *pluvialis* genomes, and full-length cDNAs of seven cryptochrome-encoding genes were cloned (Figure 2A). Two cryptochrome genes were identified in *P*. *tricornutum* and named *PtCPF1* (Gene ID: 7201137) and *PtCPF2* (Gene ID: 7199524). Meanwhile, five cryptochrome genes were identified in *H*. *pluvialis* and named *HpCRY1*, *HpCRY2*, *HpCRY3*, *HpCRY4*, and *HpCRY5* (their sequences were in Supplementary Table S5) according to the *H*. *pluvialis* transcriptome data.

Based on the phylogenetic analyses, these seven cryptochromes can be divided into three types, animal-like cryptochrome, plant cryptochrome, and DASH-type (*Drosophila*, *Arabidopsis*, *Synechocystis*, *Homo*) cryptochrome (Figure 2B). HpCRY2, HpCRY3, and PtCPF1 have high sequence homology with animal-like cryptochrome, which evolved from (6–4) photolyase and always acts as major regulators of circadian rhythms (Todo, 1999; Coesel et al., 2009; Heijde et al., 2010; Zou et al., 2017). Therefore, it is speculated that these three cryptochromes may have similar modes of action, and act as blue light receptors to regulate cell physiology and metabolism. HpCRY1, HpCRY5, and PtCPF2 are found to be closely related to DASH-type cryptochrome. However, the DASH-type cryptochromes exhibit a variety of functions in different species and the specific function is still unclear. HpCRY4 is the only cryptochrome that is classified to plant cryptochrome, and the function of HpCRY4 may be more similar to the plant cryptochrome which is involved in various growth processes such as photomorphogenesis and circadian regulation.

Compared to cryptochromes in the model plant *Arabidopsis thaliana*, there is less research on microalgal cryptochromes. Although cryptochromes from *P*. *tricornutum* and *H*. *pluvialis* were reported, respectively, in previous literatures (Petersen et al., 2021), we first dug more cryptochromes encoding genes at once and classified them into all cryptochromes types *via* bioinformatics. And it can be seen that the biological functions of algal cryptochromes are remarkably diverse among algal species. The function prediction of algal cryptochromes has certain references, but the molecular mechanisms of the relationship between cryptochrome and carotenoid biosynthesis need further analysis.

### Blue light stimulates the expression of *HpCRY4* and *PtCPF1*

To validate the connection between cryptochrome and carotenoid biosynthesis in microalgae, the cryptochrome gene expression levels under white and blue lights were acquired using qRT-PCR. According to the types of cryptochromes, two cryptochrome genes from each microalga were selected for transcript abundance quantification. Among them, *HpCRY1* and *HpCRY4* were selected from five cryptochrome genes of *H*. *pluvialis*.

**Figure 3:**
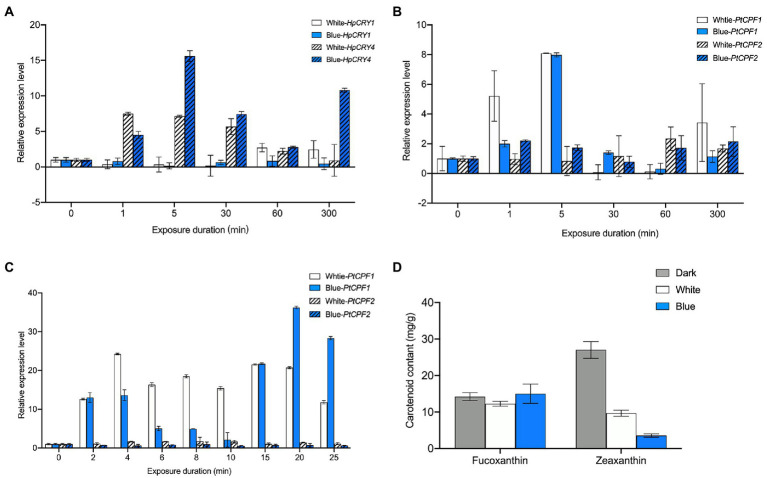
Expression analysis of cryptochrome genes under different light irradiations. **(A)** The transcription level of *HpCRY1* and *HpCRY4* in *H*. *pluvialis* in 300 min. The expression level of *PtCPF1* and *PtCPF2* in *P*. *tricornutum* in 300 min **(B)** and 30 min **(C)**. **(D)** Fucoxanthin and zeaxanthin content in *P*. *tricornutum* under different light irradiation after 30 min. The vertical bars are the means ± SD of three biological replicates.

In *H*. *pluvialis*, the transcription level of *HpCRY4* under both light irradiations increased initially and then decreased (Figure 3A). However, compared with that under white light, the transcription level of *HpCRY4* has a more significant improvement under blue light, which was consistent with the accumulation pattern of carotenoid content. Therefore, it is speculated that *HpCRY4* probably takes part in the modulation of carotenoid-related metabolic processes and promotes astaxanthin and zeaxanthin biosynthesis in *H*. *pluvialis*. Meanwhile, there was no significant difference in the expression level of *HpCRY1* under different light, it seems that *HpCRY1* cannot respond to light changes at the transcriptional level.

In *P*. *tricornutum*, there was no statistical significance in the transcription level of *PtCPF2* under different light irradiation in 300 min (Figure 3B). The transcription level of *PtCPF1* increased and then decreased under both light irradiations in 300 min. However, the transcription levels of *PtCPF1* under white and blue light irradiation were quite close at our sampling time point of 300 min. To further analyze the expression pattern of *PtCPF1* and *PtCPF2* and their changing trends with carotenoid contents, we reduced the induction time to 30 min and evaluated the transcription level of these two genes (Figure 3C), and fucoxanthin and zeaxanthin contents in *P*. *tricornutum* under different light qualities (Figure 3D) again. In 30 min, there was a minor change in the transcription level of *PtCPF2*, which indicates that it makes no response to light quality. However, the transcription level of *PtCPF1* was upregulated, and reached the highest level at 4 min, and maintained a high level in the remaining time. Interestingly, the transcription level of *PtCPF1* increased firstly and then decreased in the first 10 min and increased again in the 10–20 min which demonstrated that the expression pattern of the *P*. *tricornutum PtCPF1* under blue light showed a certain periodicity. The carotenoid content in 30 min was quite different from that in 300 min. The fucoxanthin content under white and blue light in 30 min was 12.29 and 11.90 mg g^−1^, respectively, which were not significantly different from the dark group (14.25 mg g^−1^; *p* > 0.05). We speculated that the synthesis of fucoxanthin required a relatively complex metabolic process, and it takes time to accumulate. While the content of zeaxanthin decreased significantly after 30 min under white and blue light irradiation. Zeaxanthin would be consumed for metabolic activities in a short period when the dark-adapted *P*. *tricornutum* was exposed to white and blue light, and zeaxanthin consumption under blue light was higher than that under white light. The zeaxanthin accumulation may accrue in the subsequent phase when *P*. *tricornutum* has adapted to the light.

From the above mentioned, the plant cryptochrome gene *HpCRY4* of *H*. *pluvialis* and the animal-like cryptochrome gene *PtCPF1* of *P*. *tricornutum* can sensitively respond to the illumination variations, and their transcription levels are significantly upregulated under white and blue light irradiation. Besides, blue light has a stronger effect on *HpCRY4* and *PtCPF1* expression than white light. Although the carotenoid content is consistent with the expression patterns of *HpCRY4* and *PtCPF1* genes, whether there is a regulatory relationship between *HpCRY4* and *PtCPF1* genes and target carotenoids biosynthesis in algae requires further analysis.

### Heterologous expression and knockout of some cryptochrome genes result in altered fucoxanthin content in *P. tricornutum*

The transformation and expression platforms of *H*. *pluvialis* have been reported in the literature (Steinbrenner and Sandmann, 2006; Kathiresan et al., 2009; Sharon-Gojman et al., 2015; Yuan et al., 2019). However, the transformation system is still immature and the expression efficiency is still low, which makes it difficult for us to characterize the function of *H*. *pluvialis* cryptochrome genes. In contrast, as an extensively studied model alga, *P*. *tricornutum* has a more mature transformation system compared with *H*. *pluvialis*. Considering that astaxanthin and fucoxanthin share the upstream part of β-carotene synthesis, we tried to heterologous expression of *H*. *pluvialis* cryptochrome genes in *P*. *tricornutum* to characterize the gene function.

**Figure 4:**
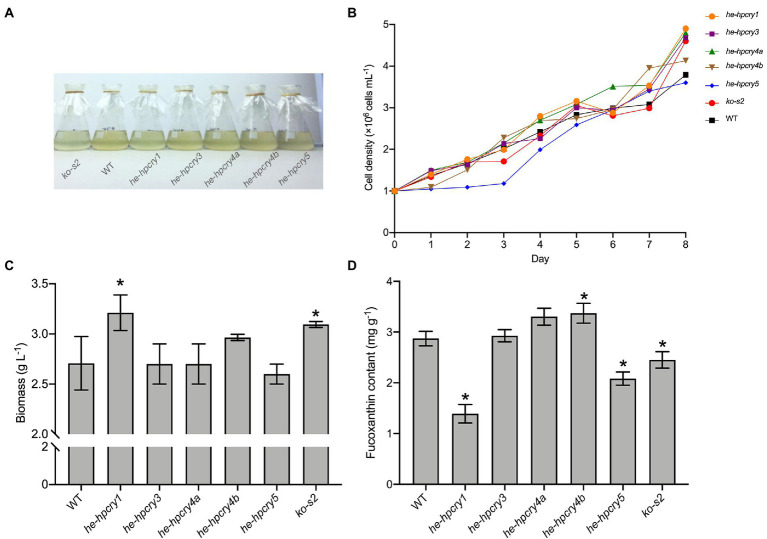
Heterologous expression and gene knockout of cryptochrome genes in *P*. *tricornutum*. **(A)** Cryptochrome genes’ heterologous expression and knockout mutants. **(B)** The growth curve of the mutants and WT. **(C)** Total biomass of the mutants and WT. **(D)** Fucoxanthin content of the mutants and WT. The vertical bars are the means ± SD of three biological replicates. Asterisks represent significant differences from the wild type (*p* < 0.05).

To further explore the connection between the cryptochrome and carotenoid biosynthesis, several heterologous expression and knockout mutants of cryptochrome genes were generated. Full-length cDNA of the *H*. *pluvialis* cryptochrome genes were cloned and ligated into the pPha-T1 vector. The complete recombinant plasmid was introduced into *P*. *tricornutum* for heterologous expression. We failed to obtain the heterologous expression mutant for *HpCRY2*, but five other *P*. *tricornutum* mutants were obtained including two heterologous expression mutants for *HpCRY4* (Supplementary Figure S2). These heterologous expression mutant strains were named *he-hpcry1*, *he-hpcry3*, *he-hpcry4a*, *he-hpcry4b*, and *he-hpcry5*, respectively (Figure 4A). Meanwhile, four sgRNAs were designed (Supplementary Table S6) to target the exon region of *PtCPF1* and one gene knockout mutant strain named *ko-s2* was generated by using the CRISPR/Cas9 genome editing system (Supplementary Figure S3). The sequence alignment with the wild-type *P*. *tricornutum* showed that thymine at position 124 was absent in *ko-s2* (Supplementary Figure S3C).

Five heterologous expression mutant strains and one knockout strain were inoculated into 250 ml flasks at an initial concentration of 1× 10^6^ cells mL^−1^. The growth curve was plotted by calculating the cell concentration every 24 h. When the mutants reached the stationary phase, their biomass and fucoxanthin contents were acquired. According to the growth curve (Figure 4B), mutant *he-hpcry5* grows more slowly than the wild-type *P*. *tricornutum* while the other mutants have quite similar growth curves to the wild-type *P*. *tricornutum*. The biomass of *he-hpcry1* and *ko-s2* reached 3.21 and 3.09 g L^−1^ which were 18.61 and 14.32% higher than that of the wild type (2.71 g L^−1^), respectively (Figure 4C; *P* < 0.05). For fucoxanthin content (Figure 4D), heterologous expression of *HpCRY1* and *HpCRY5* significantly decreased the fucoxanthin content in *P*. *tricornutum*. The fucoxanthin content of mutant *he-hpcry1* and *he-hpcry5* were 1.39 and 2.08 mg g^−1^ which were 51.64 and 27.53% lower than the wild type (2.87 mg g^−1^), respectively (*p* < 0.05). Knockout of *PtCPF1* also resulted in a decrease in fucoxanthin content, and the fucoxanthin content of *ko-s2* was 2.45 mg g^−1^, 14.63% lower than that of the wild type (*p* < 0.05). The fucoxanthin content of the two mutants for *HpCRY4* heterologous expression was 15 and 17.36% higher than that of the wild type.

Heterologous expression of *HpCRY1* in *P*. *tricornutum* results in high biomass and low fucoxanthin content, the function of *HpCRY1* needs further exploration. It is noteworthy that the fucoxanthin contents of mutant *he-hpcry4a* and *he-hpcry4b* both significantly increased. Previous studies have shown that cryptochrome-mediated light signaling pathways in plants and algae can affect the transcription levels of enzyme genes related to carotenoid metabolism (Coesel et al., 2009; Beel et al., 2017). Therefore, we supposed that heterologous expression of *HpCRY4* may promote the biosynthesis of upstream compounds of fucoxanthin and result in elevated fucoxanthin content in mutants *he-hpcry4a* and *he-hpcry4b*.

According to the transcription analysis, the expression level of the *P*. *tricornutum PtCPF1* under blue light was upregulated which consists with the increase of carotenoid content. Therefore, we hypothesized that a lower expression level of *PtCPF1* would cause a decrease in fucoxanthin accumulation. And in line with our theorized inference, the fucoxanthin content decreased in the *PtCPF1* knockout mutant *ko-s2*. However, it should be noted that knocking out *PtCPF1* does not completely block fucoxanthin biosynthesis, which revealed that carotenoid biosynthesis in microalgae is regulated by many other networks.

In summary, *HpCRY4* and *PtCPF1* are positively responsive to light quality and their transcription levels are positively correlated with the accumulation of carotenoids. The heterologous expression and gene knockout mutants of *P*. *tricornutum* further confirmed that they might regulate carotenoid biosynthesis in microalgae. Therefore, these two cryptochrome encoding genes can be used in transformation modification in microalgae for carotenoid accumulation in the future.

## Conclusion

In this study, fucoxanthin and astaxanthin contents in *P*. *tricornutum* and *H*. *pluvialis*, respectively, were significantly promoted by blue light irradiation. Five cryptochrome genes covering three types and two cryptochrome genes covering two types were identified from *H*. *pluvialis* and *P*. *tricornutum*, respectively. Among them, the expression level of *HpCRY4* from *H*. *pluvialis* and *PtCPF1* from *P*. *tricornutum* was stimulated by blue light irradiation, which has a positive correlation with carotenoid accumulation. Furthermore, changes in the fucoxanthin contents in the heterologous expression and knockout mutants indicated that *HpCRY4* and *PtCPF1* might be the potential blue light receptors and participate in the regulation of carotenoid biosynthesis. This study reveals that the molecular mechanisms of carotenoid biosynthesis in microalgae under blue light are dependent on cryptochrome, which plays important role in blue-light sensing systems and regulates cellular processes and can be used as candidate genes for industrial algae construction.

## Data availability statement

All data relevant for interpretation of this study are presented in the article and Supplementary material. Any further information is available from the corresponding author on reasonable request.

## Author contributions

ZZ and TH performed the experiments and analyzed the data. ZZ wrote the original draft. JS participated in the data analysis. HW designed and organized the study and polished the manuscript. All authors contributed to the article and approved the submitted version.

## Funding

This work was financially supported by the National Key Research and Development Program of China (Grant No. 2018YFA0902500) and the Shandong Taishan Scholars Program (No. tsqn202103144).

## Conflict of interest

The authors declare that the research was conducted in the absence of any commercial or financial relationships that could be construed as a potential conflict of interest.

## Publisher’s note

All claims expressed in this article are solely those of the authors and do not necessarily represent those of their affiliated organizations, or those of the publisher, the editors and the reviewers. Any product that may be evaluated in this article, or claim that may be made by its manufacturer, is not guaranteed or endorsed by the publisher.

## Footnotes

^1^ https://www.ncbi.nlm.nih.gov/

^2^ https://www.addgene.org/109219/
