# Supplementary material for: Cryptochrome-mediated blue-light signal contributes to carotenoids biosynthesis in microalgae
Source: Front Microbiol. 2022 Dec 22;13:1083387. doi: 10.3389/fmicb.2022.1083387 (PMC9813510; doi:10.3389/fmicb.2022.1083387)
Supplement: Supplementary file 1 [file Data_Sheet_1.docx]

*Supplementary Material*

Supplementary Figure 1


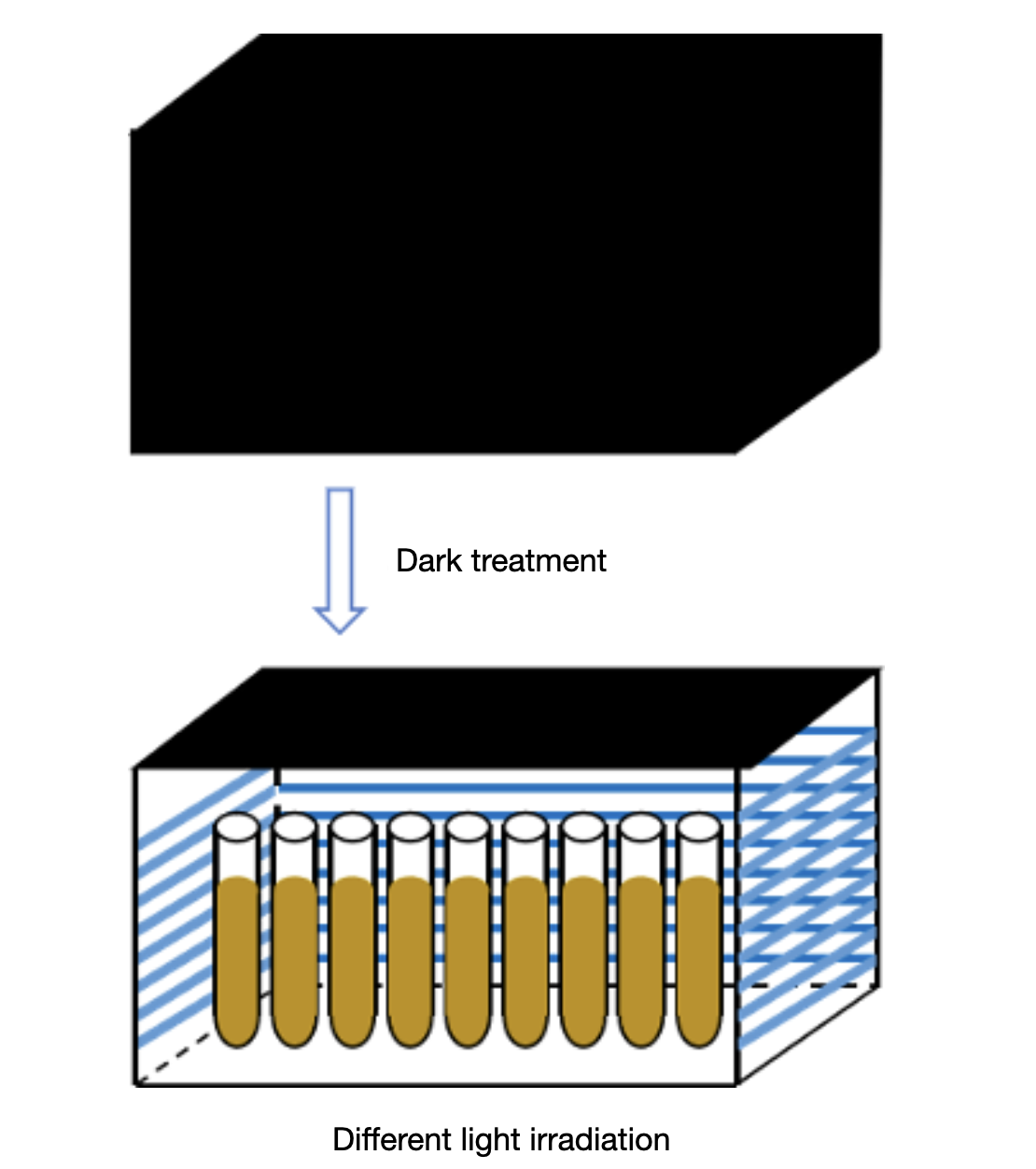


**Supplementary Figure 1 |** The cells were placed without light for 48 h for dark treatment, and then they were treated with different lights

Supplementary Table 1 | Primers used to amplify the full-length cDNA of cryptochrome genes

| Primer name | Primer sequence 5′—3′ | Annotation |
| --- | --- | --- |
| PtCPF1-F | CCCAGCTTTTAATCTATCGCTATCGTGG | amplify the full-length cDNA of *PtCPF1* |
| PtCPF1-R | CGGTTATGAATCCCATTTGTTAT | amplify the full-length cDNA of *PtCPF1* |
| PtCPF2-F | ATGAGCAGTAGCAGGTCCAAGCAGT | amplify the full-length cDNA of *PtCPF2* |
| PtCPF2-R | TTACGATTCCTTGACTCGAAAACTGCCG | amplify the full-length cDNA of *PtCPF2* |
| HpCRY1-F | ATGGACCAGACGGTGTGGGC | amplify the full-length cDNA of *HpCRY1* |
| HpCRY1-R | CATTGGCTAGTCGTCGCCAGTTGC | amplify the full-length cDNA of *HpCRY1* |
| HpCRY2-F | ATGGGTGGGGAGAGGTGCC | amplify the full-length cDNA of *HpCRY2* |
| HpCRY2-R | TCATTGGCTAGTCGTCGCCAGTTGC | amplify the full-length cDNA of *HpCRY2* |
| HpCRY3-F | ATGAGTGTGCCCAAGAATTCCATCATGTGG | amplify the full-length cDNA of *HpCRY3* |
| HpCRY3-R | CTACCTGTCGACAGTCTTAAGGCCAGAGTCCAACAATG | amplify the full-length cDNA of *HpCRY3* |
| HpCRY4-F | GGTTCACCACTGTAGCACCA | amplify the full-length cDNA of *HpCRY4* |
| HpCRY4-R | GAATTCCGACCGGCGTGTAG | amplify the full-length cDNA of *HpCRY4* |
| HpCRY5-F | GCAGATACGGGACTACTATGTGT | amplify the full-length cDNA of *HpCRY5* |
| HpCRY5-R | TTAGTGCACGACGGCTCTGCC | amplify the full-length cDNA of *HpCRY5* |

**Supplementary Table 2 |** The typical cryptochromes used for phylogenetic analyses

| Species | Name | ID |
| --- | --- | --- |
| *Homo sapiens* | *H.sapiens* CRY1 | NP_004066.1 |
|  | *H.sapiens* CRY2 | NP_066940.3 |
| *Mesocricetus auratus* | *M.auratus* CRY1 | XP_021090756.1 |
| *Drosophila melanogaster* | *D.melanogaster* CRY | NP_732407.1 |
| *Brassica rapa* | *B.rapa* CRY1 | NP_001288891.1 |
| *Armoracia rusticana* | *A.rusticana* CRY2 | BAC67176.1 |
| *Capsella rubella* | *C.rubella* CRY1 | XP_023635968.1 |
|  | *C.rubella* CRY2 | XP_023645141.1 |
| *Arabidopsis thaliana* | *A.thaliana* CRY1 | NP_567341.1 |
|  | *A.thaliana* CRY2 | NP_171935.1 |
|  | *A.thaliana* CRY3 | NP_568461.3 |
| *Oryza sativa* Japonica Group | *O.sativa* DASH CRY | XP_015644118.1 |
| *Volvox carteri* f. nagariensis | *V.carteri* DASH CRY | QIQ49260.1 |
| *Microcystis aeruginosa* NIES-88 | *M.auratus* DASH CRY | WP_061431904.1 |

Supplementary Table 3 | Primers used for qRT-PCR

| Primer name | Primer sequence 5′—3′ | Annotation |
| --- | --- | --- |
| qPtCPF1-F | TGGCAATGGCTTTCTTGCAC | detect *PtCPF1* using qRT-PCR in *P. tricornutum* |
| qPtCPF1-R | TCACCGACAATCACCCCAAC | detect *PtCPF1* using qRT-PCR in *P. tricornutum* |
| qPtCPF2-F | GTCTTTGAACTGCTGTGGCG | detect *PtCPF2* using qRT-PCR in *P. tricornutum* |
| qPtCPF2-R | TTGGCGTCTACGAGAGGGTA | detect *PtCPF2* using qRT-PCR in *P. tricornutum* |
| qPtact-F | CCAAAACCCCAAGGCGAATC | detect *β-actin* using qRT-PCR in *P. tricornutum* |
| qPtact-R | AGATCAAGACGGATGACGGC | detect *β-actin* using qRT-PCR in *P. tricornutum* |
| qHptub-F | CAACGCAGTGTTTGAGCCT | detect *α-tubulin* using qRT-PCR in *H. pluvialis* |
| qHptub-R | CAACGGCAGCATTCACATCC | detect *α-tubulin* using qRT-PCR in *H. pluvialis* |
| qHpCRY1-F | ATGGACCAGACGGTGTGGGCG | detect *HpCRY1* using qRT-PCR in *H. pluvialis* |
| qHpCRY1-R | AGCCCTGTGTGGCAGTCAAGCG | detect *HpCRY1* using qRT-PCR in *H. pluvialis* |
| qHpCRY4-F | GGTCCCATTGGCAAGTCCAT | detect *HpCRY4* using qRT-PCR in *H. pluvialis* |
| qHpCRY4-R | AGCAGGCTCTCAAACCAGTC | detect *HpCRY4* using qRT-PCR in *H. pluvialis* |

Supplementary Table 4 | Primers used to amplify cryptochrome genes for heterologous expression and primers used for verification of mutants

| Primer name | Primer sequence 5′—3′ | Annotation |
| --- | --- | --- |
| phat-Hp1-F | tgtctgccgtttcgagaattcATGGACCAGACGGTGTGGG | add *HpCRY1* to the 5′ terminal of pPha-T1 |
| phat-Hp1-R | tgcctgcaggtcgactctagaTCATTGGCTAGTCGTCGCCA | add *HpCRY1* to the 3′ terminal of pPha-T1 |
| phat-Hp2-F | tgtctgccgtttcgagaattcATGGGTGGGGAGAGGTGCC | add *HpCRY2* to the 5′ terminal of pPha-T1 |
| phat-Hp2-R | tgcctgcaggtcgactctagaTCATTGGCTAGTCGTCGCCA | add *HpCRY2* to the 3′ terminal of pPha-T1 |
| phat-Hp3-F | tgtctgccgtttcgagaattcATGAGTGTGCCCAAGAATTCCA | add *HpCRY3* to the 5′ terminal of pPha-T1 |
| phat-Hp3-R | tgcctgcaggtcgactctagaCTACCTGTCGACAGTCTTAAGGCC | add *HpCRY3* to the 3′ terminal of pPha-T1 |
| phat-Hp4-F | tgtctgccgtttcgagaattcATGGCCAGTTTGGCAGCC | add *HpCRY4* to the 5′ terminal of pPha-T1 |
| phat-Hp4-R | tgcctgcaggtcgactctagaTCATCAGCCATGGCTCGTG | add *HpCRY4* to the 3′ terminal of pPha-T1 |
| phat-Hp5-F | tgtctgccgtttcgagaattcATGCATCTGCAGATACGGGACT | add *HpCRY5* to the 5′ terminal of pPha-T1 |
| phat-Hp5-R | tgcctgcaggtcgactctagaTTAGTGCACGACGGCTCTGC | add *HpCRY5* to the 3′ terminal of pPha-T1 |
| test-Hp1-F | ATGGACCAGACGGTGTGGGC | detect *HpCRY1* in resistant transformants |
| test-Hp1-R | CATTGGCTAGTCGTCGCCAGTTGC | detect *HpCRY1* in resistant transformants |
| test-Hp2-F | ATGGGTGGGGAGAGGTGCC | detect *HpCRY2* in resistant transformants |
| test-Hp2-R | TCATTGGCTAGTCGTCGCCAGTTGC | detect *HpCRY2* in resistant transformants |
| test-Hp3-F | TTTGAGAAACCAGCCACGGA | detect *HpCRY3* in resistant transformants |
| test-Hp3-R | TGTACACCCGGAAGTATGCG | detect *HpCRY3* in resistant transformants |
| test-Hp4-F | TACAGGGTAACGGTGGACCT | detect *HpCRY4* in resistant transformants |
| test-Hp4-R | TTTGTGCTGTAGTCCCCACC | detect *HpCRY4* in resistant transformants |
| test-Hp5-F | ATGTGTTCACAGCCATGAGGT | detect *HpCRY5* in resistant transformants |
| test-Hp5-R | GCAAAGTACTGCCAGTTGACG | detect *HpCRY5* in resistant transformants |

**Supplementary Table 5 |** Sequences of the full-length cDNA of *HpCRY1*, *HpCRY2*, *HpCRY3*, *HpCRY4*, and *HpCRY5*

| Name | Sequence 5′—3′ |
| --- | --- |
| *HpCRY1* | ATGGACCAGACGGTGTGGGCGGGTCACTTCGGCACCCTCACTCCTTTCCTCAAGGCCTGGGAAAAGCTTGGCCCTATTCCTGCCCCTGTGAAGCCGCCTAACCACCTGCCTGTGGCTCCCGGCTCCCCAGCAGCCCTGTGTGGCAGTCAAGCGCTGGCGCTGTACCGCCCGGCCGTCAAGGCCGACACTGGTGAGAGCATCGACTGGGCGGCGCCGATCCGCGCCACCTGGGACATGAGTGAGGCCGCGGCGTTACGAGAGCTGGATCGATTCCTAGCTGAGGGCTTGTTGCAGTACGAGAAGAACAGGCACCTTGCGGATGCAAGAGCTGTCTCCCGGCTGTCGCCGTACCTTCGCTGGGGCCAACTCAGTCCGCGGCTGCTGTGGCACCGCATGCGCGAATCGCGGGCCGCGTCTGTCTCCAAAACCTTCCACCGGCGCTTGGTGTGGCGCGAGCTGGCTTACTGGCAGCTGCATCATTGGCCTCTGCTGTCGGTCCAGCCAGTACGGGAGGCTTATGCCGACGTGGCGTGGGAGTCTGGGCCCCAGGCTGACGAGCTGCTGGCCGCCTGGCAGCAGGGCCGCACCGGCTTCCCTCTGGTGGATGCAGGCATGCGTGAGCTGTGGCACACCGGGTGGATGCAGCAGAGCCTGCGCATGGTGTGCGCCGCCTTCCTCACTGAGCTGGCTGGCCTGCCCTGGACTGCTGGCGCCGCCTGGTTCCACCACACCTTGGTGGATGCTGACCCCGCCATCAACAGCATGATGTGGCAGAATGCTGGCAAGTCGGGTCTTGACCAATGGAACTTCAGCATGGACCCAGTTGGCAAAGCGCAGGACCCTCAAGGGCAGCACATCAGAAAGTGGGTGCCAGAGCTGGCTAAGCTGCCTGACGCTCACGTGCACACGCCCTGGCTTGCCCCACCTGACGTGCTGGAGCAGGCTGGGGTGCATCTGGGCCGGACCTACCCACATCGCGTGTTGCAAGGGAGCCTACAGGCCGCTAAGGCGCAAGGGTTAGCCAAGCTGCAAGCCGCCCGTGACTTGCATGCCGAAACATGGTGTGACGCGCGAGGTTACGACCTGGTCATTGCGCCCAAGGGATCCTCAGTGGGTCTGGACGGCAAGAAGGTGGTGGTGTTTACAATCCCTGAACTCCGGGCCGCCACCGCGACGAAGTCAATCATCGACGTAGCTTTAAAGCTGAAGCCTGAGCAGCAACTGGCGACGACTAGCCAATGA |
| *HpCRY2* | ATGGGTGGGGAGAGGTGCCCTGCTCGGCGTGCCAGCCTTTGCGCCCATACCGACCGTCCATTGCGTTATCGTGCCTCGTGCCCGCACCCACTCGATCCTGTCGCAGCGCTGGCGCTGTACCGCCCGGCCGTCAAGGCCGACACTGGTGAGAGCATCGACTGGGCGGCGCCGATCCGCGCCACCTGGGACATGAGTGAGGCCGCGGCGTTACGAGAGCTGGATCGATTCCTAGCTGAGGGCTTGTTGCAGTACGAGAAGAACAGGCACCTTGCGGATGCAAGAGCTGTCTCCCGGCTGTCGCCGTACCTTCGCTGGGGCCAACTCAGTCCGCGGCTGCTGTGGCACCGCATGCGCGAATCGCGGGCCGCGTCTGTCTCCAAAACCTTCCACCGGCGCTTGGTGTGGCGCGAGCTGGCTTACTGGCAGCTGCATCATTGGCCTCTGCTGTCGGTCCAGCCAGTACGGGAGGCTTATGCCGACGTGGCGTGGGAGTCTGGGCCCCAGGCTGACGAGCTGCTGGCCGCCTGGCAGCAGGGCCGCACCGGCTTCCCTCTGGTGGATGCAGGCATGCGTGAGCTGTGGCACACCGGGTGGATGCAGCAGAGCCTGCGCATGGTGTGCGCCGCCTTCCTCACTGAGCTGGCTGGCCTGCCCTGGACTGCTGGCGCCGCCTGGTTCCACCACACCTTGGTGGATGCTGACCCCGCCATCAACAGCATGATGTGGCAGAATGCTGGCAAGTCGGGTCTTGACCAATGGAACTTCAGCATGGACCCAGTTGGCAAAGCGCAGGACCCTCAAGGGCAGCACATCAGAAAGTGGGTGCCAGAGCTGGCTAAGCTGCCTGACGCTCACGTGCACACGCCCTGGCTTGCCCCACCTGACGTGCTGGAGCAGGCTGGGGTGCATCTGGGCCGGACCTACCCACATCGCGTGTTGCAAGGGAGCCTACAGGCCGCTAAGGCGCAAGGGTTAGCCAAGCTGCAAGCCGCCCGTGACTTGCATGCCGAAACATGGTGTGACGCGCGAGGTTACGACCTGGTCATTGCGCCCAAGGGATCCTCAGTGGGTCTGGACGGCAAGAAGGTGGTGGTGTTTACAATCCCTGAACTCCGGGCCGCCACCGCGACGAAGTCAATCATCGACGTAGCTTTAAAGCTGAAGCCTGAGCAGCAACTGGCGACGACTAGCCAATGA |
| *HpCRY3* | ATGAGTGTGCCCAAGAATTCCATCATGTGGTTTAGAAAAGGGTTGCGCTTGCACGACAACCCCGCGCTGCTAGAGGCATGCAAAGGGGCGACCCATATGTTCCCCGTCTTCATCATCGACCCCTTTTTTCTCCAGAACCCAACAGCCTACAAGGTGGGTGTTAACCGCTACAACTTCCTCCTTGAGAGCCTGAATGACCTGGATCAAAGCTTCAGGGCACGAGGCAGCCGGCTGCTGGTGGTCCGCGGCAAGCCGGTGGAGGTGCTGCCACGCCTGATGAAGGACTGGGCCATCACCCAGCTGTGCTTCGAGGTGGACACGGAGCCGTATGCAACACAGCGGGATGCCCAGGTGCAGCAGCTGGCCGCTGAGGCTCGGGTGGAGGTCAGGGCCTTTGTGAGCCATACGCTGTATGACACTGCCCAGCTGGTCACCCGCAACCAGGGCAAGGCTCCCACCACCATGACCAGCTTCTGCAAGCTGCTGGACAAAGTGGGTGACCCGCCCGCGCCGGCCCCGGCCCCCCCACAGCAGCTGCCGCCAATCCCAGCAACCGCCCCAGGGGCCGAGGAGGAGGCCACCGGGGTGCCCTGCTGCCTGGACATGGGCTTTGACAGGCCGCCCTCCACCATATTCAAGGGTGGGGAGACAGAGGCACTCAAGCGCCTGGAGAGAGCGTTTGCTGACCCGCAGTGGATCGCCAAGTTTGAGAAACCAGCCACGGACCCCTCAGCCTTCCAGCAGCCCGCCACCACTGTCCTGTCCCCGTACCTCAAGTTCGGCTGCGTGTCCGCACGACTCTTCCACCAACGCCTGCTGCAGGTGTACGCCACTCACACCGGGCACAGCCAGCCGCCCATGTCCCTGCGTGGGCAGCTGCTGTGGCGCGAGTTCTTCTACACAGTGGGGGCGCACACCCCCAACTTCTCCGCCATGGCAGGCAACCCGCTGTGCCGCCAGATCCCCTGGCAGAGCAACCCGCAGCACCTGGCAGCCTGGAGGGAGGGGCGCACTGGCTTCCCCTGGATTGACGCCATCATGGTGCAGCTCAACACCTGGGGCTGGATGCACCACCTTGCGCGGCACAGCGTGGCCTGCTTCCTCACCCGGGGTGACCTGTACCTCAGCTGGGAGGAGGGGCAGGCGGTGTTTGAGGAGCTGCTGATCGACCAGGACCACTTCATCAACGCGGGCAACTGGATGTGGCTGAGCGCCAGCGCCTTCTTCTCCGCATACTTCCGGGTGTACAGCCCAATCACCTTTGGCAAGAAGTACGACAAGGAGGGCGTGTTCATCCGCAAGTTCCTGCCCGTGCTCAAGGACATGCCGGCCAAGTACATCTATGAGCCCTGGACAGCCCCTCTGGATGTGCAGCGCAAAGCAAAGTGTGTGGTGGGCAAGGACTACCCCTCACCCATTGTGGACCATGCGGTGGTCAGCAAGGCAAACATCGCCCGTATGGCCGCAGCCTACAAGGCAGGGTCCATGCCCGCACATGACGACGGGGGTGACGAGCCTGAAAACGGACCGGCCCGGGCTGGCGCACGGAGCGGGTCCGGCAGTCGCAAGGCAGCCTCGTCACCCGGCAAGCCGGTTAAGAGCATCGCGTCGCTACTGGCTGACGCGAGCAAAGCTCGACCCGCCGCGGACCGTGTTGAGAGCCAGGCGGCTGTGGTGCCGCGTGCGGCGCGGCCCGTGCAACAGACCTTAGAGCAGACTATGGCGGGCAGAGGCTCGTCCCGAGACGTGCCCGAGATCAAAGTCGAAGACAGTAAAGGCCTTCACGGCGTAGCTGACGCAGCGAGAATGCAGGCTGGGGCTGAGGAGTCAGATTTGGGTGCCGGCACAAGCGTCGTGCGTCTTGTAACGCCAGCTGTGCCGGTAGAGAACGGTGGGGCACCCTCTCTTCCGGCCGCCAGAGTGCGGAAGGTCGGGGCCGCAGCGAAGTCAGTCATGAAAGGCAGCGTGGCGAGTCCTTCCCCGGGTAGCTCGTCAAAGCGCCAGCGCACATTGTTGGACTCTGGCCTTAAGACTGTCGACAGGTAG |
| *HpCRY4* | ATGGCCAGTTTGGCAGCCAGTGTAGCGTCAGGAGCGCCTGCAGTGCGGCAACCAGGGAGCACACGCCGTCTGCTCTTGTGGTTTCGCAATGACCTGCGGGTTCACGACAACTACACCATCAGTGAGGCGGCTCGTCTCATCAAGTCACGGCAGTTTGCAGAGGTGCTGCCGGTGTATGTGTTTGACCCCCGCAACTTCATCATCACCCCGTTTGGCAACCCCAAGACTGGCGGTCACAGGGCGCAGTTCCTGCTGGAGAGCGTGGCTGACCTGAAACAGCAGCTGCGAAGACTGGGCAGCGACCTAGCCGTGACGGTGGGCAAACCAGAGCACATCCTCCCAGGCCTGCTGATGGGCTGCCCTAGCTCCTTGGTGCTGACTATGGAGGAGTCCACCAGCGAGGAGAAGACGGTGGACGCTGCGGTGGTCAAGGCGCTGGACCCTGCAGGGTCACGGCTGCAGAAGGTGTGGGGTCACACTCTGTATCACCTGGATGACCTACAGGGTAACGGTGGACCTTTCAGCTCTGGAGGCATCAAGACCATGCCTAACATCTTCACACCCTTCAAGGAACAGGTGGAGAGGCGCTGCCAGGTACGGGCCTTAGCACCCACTCCCGGCCTGGGCTCGCTGCCGCTGCCTGACCCTGCAGCCCCTGTGGCCTTCCCTGGCAACAGCTGTCACAGCAGCAGCAGAGGGGGTGGTGGTGCTGAAGGGGCGGGCAGTGTGGGGCAGCTGTTGGCCTGGGAGGCTGACTGGGCCAGTCTGCCCTGGCCAGATGTCTTCAAGCCAAAGGCTCCGCCTGCAAAGGACCCCAGAGCAGTGTTAGACTACCGCGGTGGTGAGACGGTGGCTCTAGCACGCCTGAAGTATTACCTGTGGGATTCAGACCTGCTGGCCACATACTTTGACACGCGTAACGGGATGCTGGGTGGGGACTACAGCACAAAGTTCAGCGCCTGGATGGCAGCCGGCTGCCTGTCTCCACGTCAGGTCTACCACGAGATCAAGCGCTATGAGCAGCAGCGCACCGCAAACAAGTCGACCTATTGGGTGGTGTTCGAGCTGCTGTGGCGGGACTTCTTTCGCTTCTACGCCATCAAGCATGGCTCGCGCATCTTCTTTGAGACTGGTCCCATTGGCAAGTCCATTCCGTGGAGTGCTGACCCTAGCCTGCTGCAGCGCTGGCAGGAGGGGCGCACTGGCATGCCCCTGGTGGACGCCAACATGAGGGAGATTGCAGCCACGGGCTTCATGAGCAACCGAGGCAGGCAGAACGTGGCGTCCTACTTGGTGCTGGACCTGGGCGTGGACTGGCGCAAGGGTGCTGACTGGTTTGAGAGCCTGCTGCTGGACCACGACGTGACCAGCAACTGGGGCAACTGGGTAGCGGCTGCAGGCCTCACGGGGGGACGCATCAATGCCTTCAACATCACCAAGCAGAGCAAGGACTATGACCTGATGGGCGAGTACCTGCGCACTTGGCTGCCGGAGCTGGCCAAGGTGCCGGCGCCTCACATCCACGAGCCATGGCTGATGAGCAAACAGGACCAGGACAAGTATGGGGTTCAAATAGGCGTGGATTATCCGGCACCTGTGCCAGCGTCGCAACTGGCCCGACCCCACTCCGCCTATGGCGCATCACGAGGCAATGGCAGGCCCTACAGCGGCCGCGGGAGCCGAGACGACAACGGGCCCGGGCGAGGGGGTGGAGTCGGACGCGGTGCTGGCGGTCGGGGCGGTCGGGGGCAGCCTACACGCCGGTCGGAATTCGAGCGATTCGGGTAG |
| *HpCRY5* | ATGCATCTGCAGATACGGGACTACTATGTGTTCACAGCCATGAGGTCAGGGCCAGCTCTCATGGCCCCGGGGGGGCTGCAGGGGGGCAGCAGCCACTGGCGCCACGACATGCCTGCCTTCACGCGGTGGGCGCGGGGTCAGACGGGGCTGCCCTACGTGGACGCCTCCTTGAGGGAGCTTGCCGGCAGCGGCTGGTTGAGCAACAGGGGGCGGCAGAACGTGGCCAGCCTGCTGACCAAGGAGCTGGGGCTGGACTGGCGGCTAGGCGCTGCCTGGTTCGAGTCGCTGCTGGTGGACCACGACCCCGCCGTGAACGCCGTCAACTGGCAGTACTTTGCGGGGGTGGGCACTGACCCGCGCAACCGGCGCTTCAAGACCGTGTCGCAGGGCATGCAGTACGACGAGGACGCAGTGCTGGTCAAGCTGTGGGTGCCCGAGCTGGCCAGCCTGCCCCCGGCCCTGCGCCACGCGCCCTGGCTGGCGTCAGCGGAGGAGGCCAGGGCGGGCGGGCTGCAGCTGGGGGTGACCTACCCCACAGCCATGGTGGACCCTGCGGGCCAGACAGGCGCCCTGCCGGCTGCAGCCAAGGCAGGCGGCAAGCAGGCAGGCCACAAGAGCAGCCGGGTGGCTGAGGGGCTGGGGGAGGCGCTGGCCCGCAAGCCGGAGAAAGAGGAAGAAGAGTGCTCCGGCAGAGCCGTCGTGCACTAA |

Supplementary Figure 2


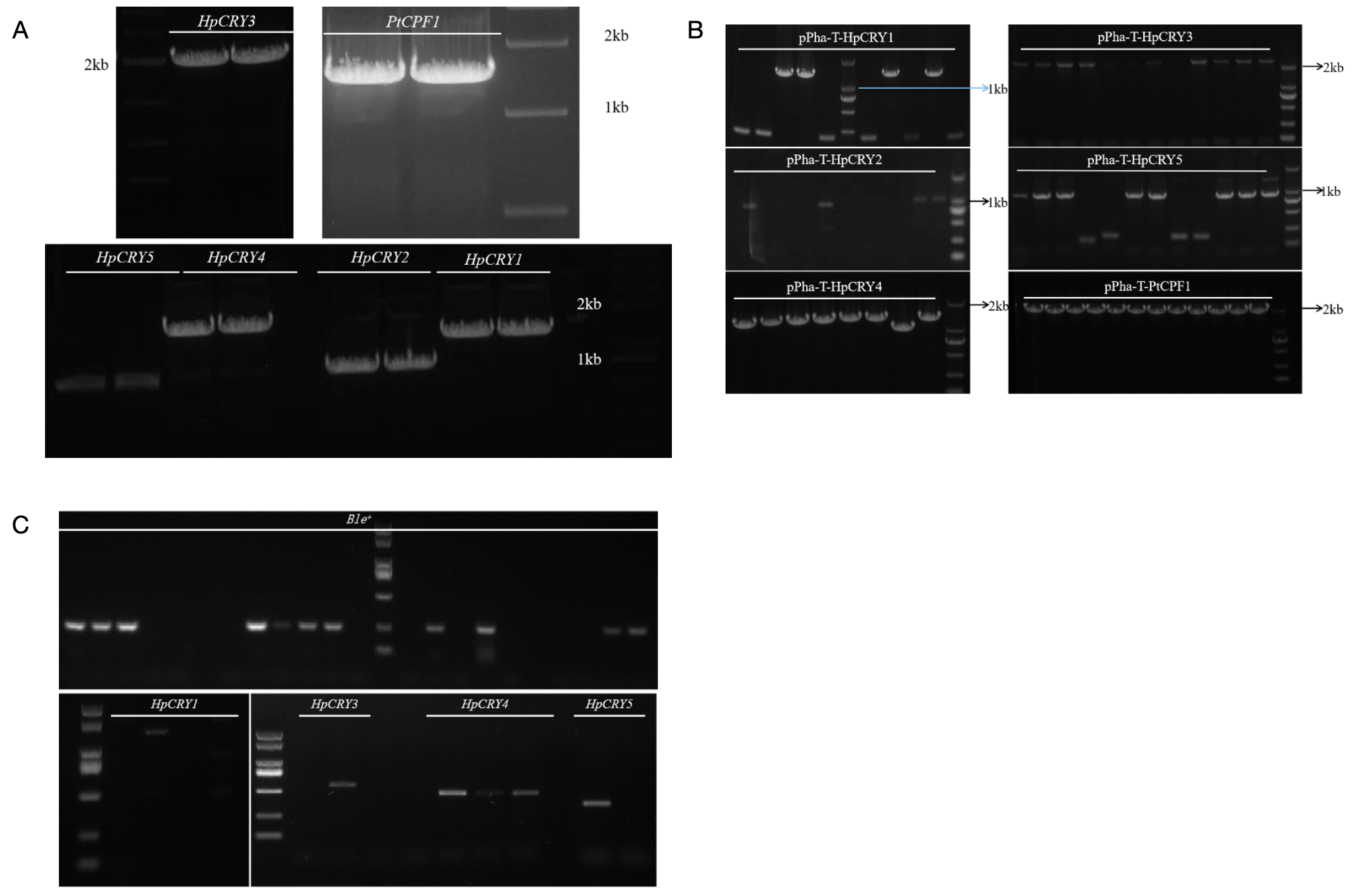


**Supplementary Figure 2 |** (A) Full-length cDNA cloning of the cryptochrome genes from *H. pluvialis*. (B) PCR verification of vectors construction for heterologous expression. (C) PCR verification of mutants using primers specific for the heterologous cryptochrome genes.

**Supplementary Table 6 |** Four gRNAs designed to target the exon region of *ptcpf1*

| Name | sequence of gRNA 5′—3′ | Annotation |
| --- | --- | --- |
| sg1-F | TCGAGTAAATCGGAAGGAGTGATT | to form double-strand gRNA |
| sg1-R | AAACAATCACTCCTTCCGATTTAC | to form double-strand gRNA |
| sg2-F | TCGAGTCAGGGTCGACTACGTAAAT | to form double-strand gRNA |
| sg2-R | AAACATTTACGTAGTCGACCCTGAC | to form double-strand gRNA |
| sg3-F | TCGAGTTCCCTTCGCGCAAACTGCT | to form double-strand gRNA |
| sg3-R | AAACAGCAGTTTGCGCGAAGGGAAC | to form double-strand gRNA |
| sg4-F | TCGAGCAATGCCTTAGCCTGTGTGG | to form double-strand gRNA |
| sg4-R | AAACCCACACAGGCTAAGGCATTGC | to form double-strand gRNA |
| Test-sg-F | AAGTCTTGGACGCCACCCTC | primers used to verify the recombinant plasmid |
| M13R | CAGGAAACAGCTATGAC | primers used to verify the recombinant plasmid |

Supplementary Figure 3


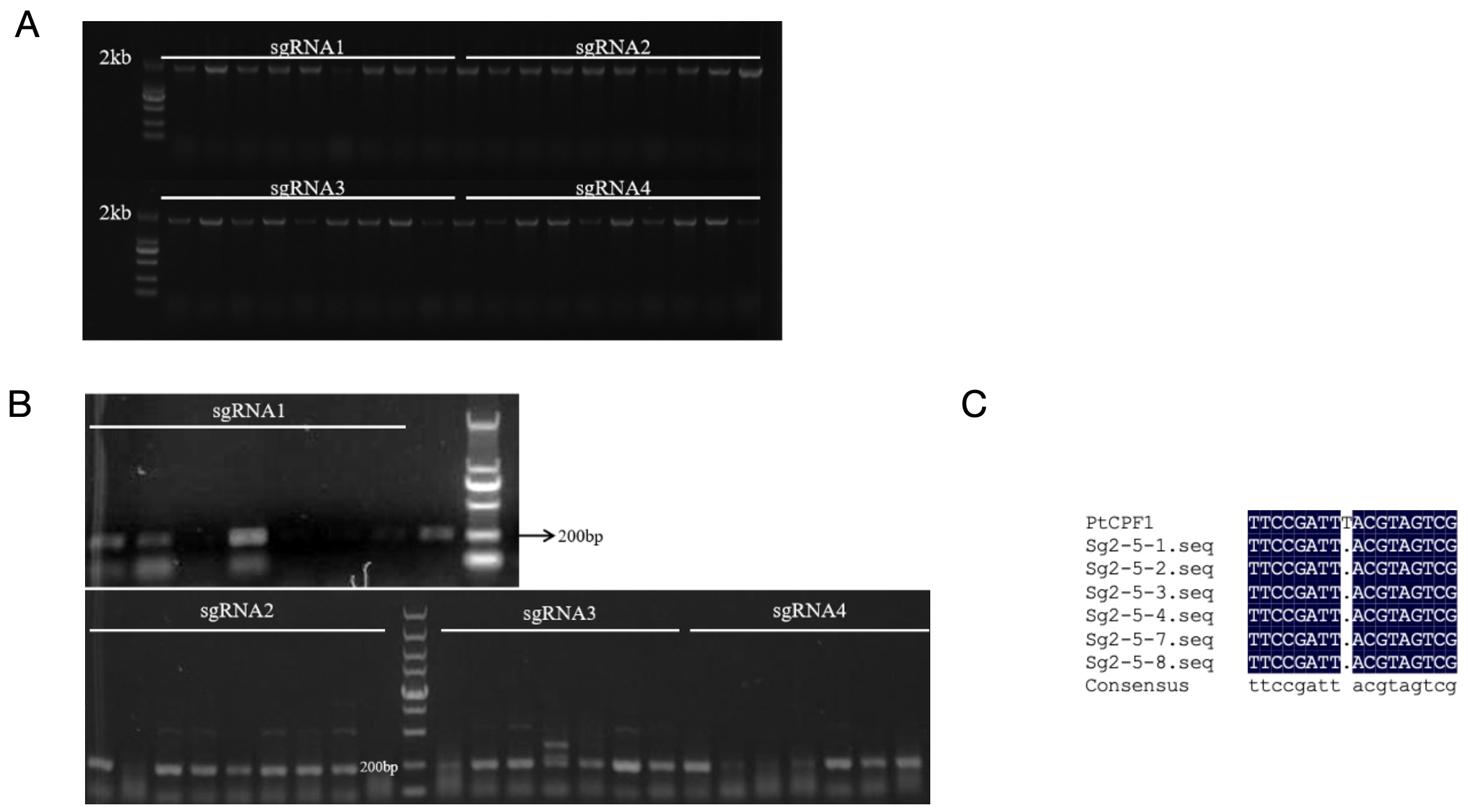


**Supplementary Figure 3 |** (A) Colony PCR examination of pPtpuc3-diaCas9 knockdown plasmids. (B) PCR examination of transformants by exogenous cryptochrome gene specific primers. (C) Sequence alignment of *PtCPF1* gene from mutants and wild type.
